# The severity and atypical presentations of COVID-19 infection in pediatrics

**DOI:** 10.1186/s12887-021-02614-2

**Published:** 2021-03-25

**Authors:** Nagwan Y. Saleh, Hesham M. Aboelghar, Sherif S. Salem, Reda A. Ibrahem, Fatma O. Khalil, Ahmed S. Abdelgawad, Asmaa A. Mahmoud

**Affiliations:** 1 Department of Pediatrics, Faculty of Medicine, Menoufia University, Shebin Elkom, Egypt; 2 Department of Public Health and community Medicine, Faculty of Medicine, Menoufia University, Shebin Elkom, Egypt; 3 Department of Clinical and Molecular Microbiology and Immunology, National Liver Institute, Menoufia University, Shebin Elkom, Egypt; 4 Department of Clinical Pathology, National Liver Institute, Menoufia University, Shebin Elkom, Egypt

**Keywords:** Children, COVID-19, Hypoxia, Mechanical ventilation, Shock

## Abstract

**Background:**

Emergence of 2019-nCoV attracted global attention and WHO declared COVID-19 a public health emergency of international concern. Therefore we aimed to explore the severity and atypical manifestations of COVID-19 among children.

**Methods:**

This is an observational cohort study conducted on 398 children with confirmed COVID-19 by using real-time reverse transcriptase polymerase chain reaction assay for detection of 2019-nCoV nucleic acid during the period from March to November 2020. Patients were subdivided regarding the severity of COVID-19 presentation into Group I (Non-severe COVID-19) was admitted into wards and Group II (Severe COVID-19) admitted into the PICU.

**Results:**

Non- severe cases were 295cases (74.1%) and 103cases (25.9%) of severe cases. There was a significant difference between age groups of the affected children (*P* < 0.001) with a median (0–15 years). Boys (52%) are more affected than girls (48%) with significant differences (*P* < 0.001). 68.6%of confirmed cases had contact history to family members infected with COVID-19. 41.7% of severe patients needed mechanical ventilation. Death of 20.4% of severe cases. In COVID-19 patients, fever, headache, fatigue and shock were the most prominent presentations (95, 60.3, 57.8, and 21.8% respectively). 3.5% of children were manifested with atypical presentations; 1.25% manifested by pictures of acute pancreatitis, 1.25% presented by manifestations of deep venous thrombosis and 1.0% had multisystem inflammatory syndrome (MIS-C). Multivariate regression analysis showed that COVID-19 severity in children was significantly higher among children with higher levels of D-dimer, hypoxia, shock and mechanical ventilation.

**Conclusion:**

Most children had a non-severe type of COVID-19 and children with severe type had higher levels of D-dimer, hypoxia, shock and mechanical ventilation.

## Introduction

In Wuhan, Hubei province, China, many cases of pneumonia of unknown cause emerged in December 2019 with rapid spread [1]. Enveloped RNA coronavirus has been identified in bronchoalveolar lavage fluid samples taken from these patients [2, 3].

Initial name of this new coronavirus was 2019-nCoV. Later on, it was termed severe acute respiratory syndrome coronavirus 2 (SARS-CoV-2) [4]. This virus is a member of respiratory tract viruses group which is related to Middle East respiratory syndrome coronavirus (MERS-CoV) and severe acute respiratory syndrome coronavirus (SARS-CoV). The World Health Organization (WHO) termed it as coronavirus disease 2019 (COVID-19) [5].

Pandemic SARS-CoV-2 was transmitted from human to human through respiratory droplets by coughing and sneezing or direct contact resulting in worldwide outbreak [6]. The virus might be transmitted through fecal-oral route particularly in infants and children who are not toilet trained [7].

Adult patients with COVID-19 present with fever, dry cough, dyspnea, fatigue, and lymphopenia. Severe pneumonia is more common in elderly adult patients and those with chronic comorbidities, having greater risk for developing severe acute respiratory syndrome and even death [8]. Reports of children with COVID-19 are increasing [9–13]. So the aim of the present study was to explore the severity and atypical manifestations of COVID-19 among children.

## Methods

### Design

This is an observational cohort study conducted on 398 children at four hospitals in Shebin Elkom city, Menoufia Governorate, including (Menoufia University Hospital and Shebin Elkom Teaching Hospital, Shebin Elkom Chest Hospital, El-Helal Insurance Hospital), Egypt during the period of first of March 2020 to the last November 2020. We included all children (aged 0–18 years) with laboratory confirmed COVID-19. Three hundred ninety-eight cases were confirmed by using real-time reverse transcriptase polymerase chain reaction assay (PCR) for detection of 2019-nCoV nucleic acid.

COVID-19 diagnosis was based on clinical manifestations and contact history regarding WHO and recommendations for diagnosis, prevention and control of the 2019 novel coronavirus infection in children [14, 15] .Within the last 14 days, if a child was exposed to a COVID-19 case, patient considered as high risk or lived in an epidemic area; a community where a COVID-19 case was reported defined as having medium risk, or lived a non-epidemic area where no COVID- 19 case was reported, defined as having low risk. So, suspected children were identified if a child at high risk had two of the following conditions: (1) Fever, respiratory, gastrointestinal symptoms (e.g., vomiting, nausea, and diarrhea), or fatigue; (2) White blood cell count was normal, decreased, or had lymphopenia or increased level of C-reactive protein (CRP); or (3) Abnormal chest radiograph imaging result. For a child at medium or low risk, similar diagnostic criteria were applied after excluding influenza and other common respiratory infections. Suspected cases were defined as confirmed cases when nasal and pharyngeal swab specimens tested positive for 2019-nCoV nucleic acid by using real-time reverse transcriptase PCR assay [15].

Patients were subdivided regarding to COVID-19 severity presentation into two groups; Group I (Non-severe COVID-19) admitted into wards and Group II (Severe COVID-19) admitted into the pediatric intensive care unit (PICU).

Criteria for severity of the disease and PICU admission included (1) The need for invasive or noninvasive mechanical ventilation, (2) Impending respiratory failure, (3) SPO_2_ < 92% on inspired oxygen> 50%, (4) Signs of shock, and (5) Altered mental status [16].

The primary outcome measure was development of any indicator of COVID-19 severity, such as hypoxia, shock, sepsis or death, within 30 days.

All parents of included children gave their written consent to participate in the study. The study was approved by the Menoufia University Ethics Committees.

### Laboratory and radiological investigations

A complete workup was performed including history taking and physical examination for all patients. The vital signs and oxygen saturation were monitored for all patients. Sepsis was defined according to the criteria established by the international pediatric sepsis consensus conference [17]. A patient was considered as having sepsis if there was evidence of SIRS (Systemic Inflammatory Response Syndrome) in the presence of suspected or proven infection. Hypoxia was defined as a sustained peripheral oxygen saturation (SPO_2_) < 94% [18]. Shock is defined as a condition in which peripheral tissues and end organs do not receive adequate oxygen and nutrients. The body compensates for shock through various mechanisms, most commonly through increased heart rate to increase cardiac output [19]. Non-contrast chest Computed Tomography (CT) studies were performed for all patients on 16-slice, and 128-slice machines (Siemens medical system; Siemens, Germany) with the patients in supine position, the scan performed at the end inspiration from the base of the neck down to the diaphragm with a suitable field of view adjusting these limits, 1.25 mm slice thickness thin sections were performed in the axial view which were then transformed to a workstation for multiplanar reconstruction and analysis of the findings with the following parameters: 12 0 kV,100 to 150 mA, 0.6-mm collimation, and 1:1 pitch.

Laboratory work-up including drawing ten ml of blood samples by sterile venipuncture. Five ml of blood were delivered to a vacutainer plain test tube and serum was then separated by centrifugation at 3000 rpm for 10 min and used for analysis of CRP and Lactate dehydrogenase (LDH) by using the Cobas 6000 analyzer (c501 module) which is a photometric unit of the auto-analyzer. Analysis of serum ferritin was done by the electro chemiluminescence immunoassay “ECLIA” using Cobas 6000 (e 601 module) (Roche diagnostics- GmbH, D-68305 Mannheim, Germany).

Three ml of blood were delivered to a vacationer plastic tube containing EDTA and used for complete blood count by using Sysmex XT 1800i (Sysmex Corporation, Kobe 651–0073, Japan) Automated Hematology Analyzer which utilizes the Fluorescent flowcytometry and hydrodynamic focusing technologies.

Two mL of blood were delivered to a vacationer plastic tube containing sodium citrate (3.2%) for D-dimer determination by using (D-DI2 Tina-quant D-Dimer Gen.2) on the Cobas 6000 analyzer (c501 module) with expected value less than 0.5 μg fibrinogen equivalent/ml (ug FEU/ml).

Nasopharyngeal and oropharyngeal swabs were taken for SARS-CoV-2 RT-PCR test. Specimens were collected and stored in a collection tube with 5 mL viral transport media according to WHO recommendations (4 °C for ≤5 days and − 70 °C for longer periods).

Detection of SARS-CoV-2 was performed by using QIAGEN Real-Time PCR system (QIAGEN, GmbH, Germany). The COVID-19 specific probe is labeled with the FAM fluorophore and the internal control is labeled with the HEX fluorophore.

* RNA was extracted by QIA cube HT Extraction System (QIAGEN, GmbH) according to manufacture instructions.

PCR was detected by using Rotor Gene real-time PCR fluorescence system (QIAGEN, GmbH, Germany) as the following conditions: reverse transcription was done at 55 °C for 10 min (one cycle), followed by initial denaturation (Taq activation) at 95 °C for 2 min (one cycle), then denaturation at 95 °C for 10 s, lastly annealing and extension at 60 °C for 60 s (45 cycles).

The assay includes an internal control and positive control to identify possible PCR inhibition, to measure extraction purity and to confirm the integrity of the PCR run. To ensure PCR run validity, the Positive Control Template (PCT) should produce Cq value ≤22 in the FAM channel.

### Statistical analysis

The data was collected, tabulated and analyzed using Statistical Package for social science (SPSS) version 20, on IBM compatible computers (SPSS Inc., Chicago, IL, USA). The quantitative data was described as mean, standard deviation, median and range and compared by Student t test and Mann Whitney U test according to the data normality. Qualitative data was described as number and percentage and compared using Chi square and Fisher’s Exact tests accordingly. Binary logistic regression was used to predict independent risk factors for severe and critical COVID-19 infection, significance was considered at a *P* value (< 0.05).

## Results

**Fig. 1:**
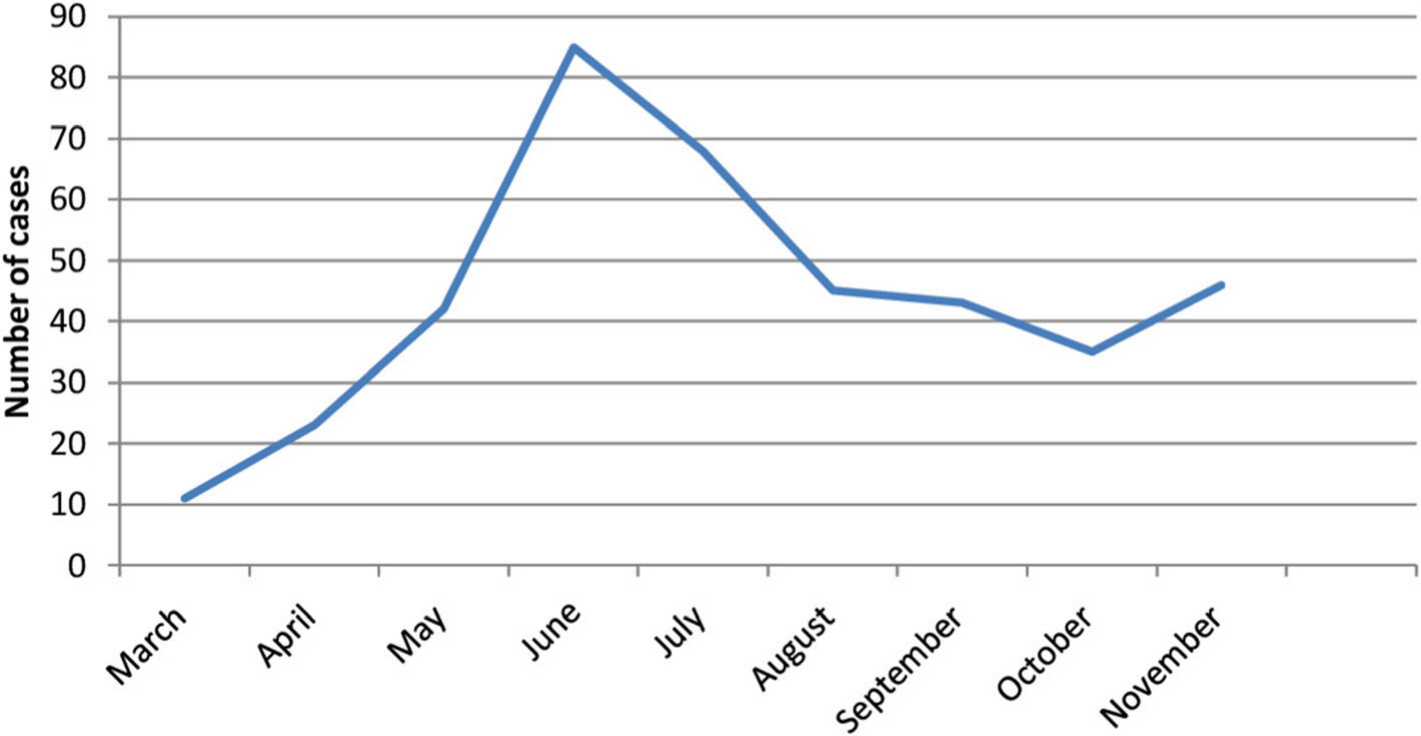
Detection of COVID-19 cases during the six-month of study period

This study was conducted on 398 confirmed cases during the period of first of March 2020 to the last November 2020 and detection of cases during the six-month of study period illustrated in (Table 1&Fig. 1).

**Table 1:** Detection of COVID-19 cases during the six-month of study period

	Number	Percent (%)
March	11	2.8
April	23	5.8
May	42	10.6
June	85	21.4
July	68	17.1
August	45	11.3
September	43	10.8
October	35	8.8
November	46	11.6
Total	398	100

Regarding to demographic and clinical data of COVID-19 children, there was a significant difference between age groups of affected children (*P* < 0.001) with a median (0–15 years). Most common affected age was less than 5 years and older than 10 years, but age from 5 to 10 years was common in severe cases. Boys (52%) are more affected than girls (48%) with significant differences (*P* < 0.001). Most of the confirmed cases (68.6%) had a contact history to family members infected with COVID-19. 41.7% of severe patients needed mechanical ventilation due to hypoxia, with death of 20.4% (*n* = 21) of severe cases (Table 2).

**Table 2:** Demographic and clinical data of the COVID-19 groups

Studied variables	COVID-19 groups		Test f sig.	*P* value
	Group I *N* = 295	Group II *N* = 103		
**Age / year**			U	
Mean ± SD	8.08 ± 5.81	8.41 ± 4.52	0.76	0.45
Median (Range)	10 (0–17)	8 (1.5–16)		
**Age groups**			*X*^*2*^	
0–5 years	126 (42.7%)	24 (23.3%)	57.1	< 0.001^**^
5–10 years	34 (11.5%)	48 (46.6%)		
> 10 years	135 (45.8%)	31 (30.1%)		
**Sex N (%)**			*X*^*2*^	
Male	162 (54.9%)	45 (43.7%)	55.4	< 0.001^**^
Female	133 (45.1%)	58 (56.3%)		
**History of contact**			*X*^*2*^	
Yes	210 (71.2%)	71 (68.9%)	0.19	0.67
NO	85 (28.8%)	32 (31.1%)		
**Family members with COVID-19** Yes	202 (68.5%)	71 (68.9%)	*X*^*2*^	
No	93 (31.5%)	32 (31.1%)	0.007	0.93
**Need for MV** N (%)			*X*^*2*^	
Ventilated	0 (0.0%)	43 (41.7%)	138.1	< 0.001^**^
Non Ventilated	295 (100%)	60 (58.3%)		
**Duration for MV(hrs**)				
Mean ± SD	–	6.5 ± 1.75	–	**–**
Median (Range)		6 (4–12)		
**Hospital stay / days**			U	
Mean ± SD	17.02 ± 8.99	16.74 ± 6.64	0.89	0.37
Median (Range)	14 (7–34)	15 (7–30)		
**Mortality**			*X*^*2*^	
**Died**	6 (2.0%)	21 (20.4%)	40.67	< 0.001^**^
**Survived**	289 (98.0%)	82 (79.6%)		

*MV* Mechanical Ventilation, *X* Mean, *SD* standard deviation, *U* Mann-Whitney U test, *X*^*2*^ Chi-square test

** highly significant

On the basis of clinical presentation of COVID-19 patients, fever, headache, fatigue and shock were the most prominent (95, 60.3, and 57.8% and 21.8% respectively), while cough, tachypnea, hypoxia and diarrhea were (39, 41.7, 34.2 and 34.7% respectively), and both rhinorrhea and sore throat were 18.3%. There was a high significant difference between two groups regarding cough, shock and hypoxia.

Fourteen children of studied patients (3.5%) were manifested by atypical presentations; five children (1.25%) manifested by picture of acute pancreatitis, five children (1.25%) presented by manifestations of deep venous thrombosis and four children (1.0%) had MIS-C (Table 3).

**Table 3:** Clinical presentation of the COVID-19 groups

Studied variables	COVID-19 groups		Test f sig.	*P* value
	Group I *N* = 295	Group II *N* = 103		
**Dry cough n (%)**	74 (25.1%)	81 (78.6%)	92.09	< 0.001^**^
**Tachypnea n (%)**	70 (23.7%)	96 (93.2%)	39.39	< 0.001**
**Rhinorrhea**	73 (24.7%)	0 (0.0%)	30.17	< 0.001^**^
**Sore throat n (%)**	73 (24.7%)	0 (0.0%)	31.21	< 0.001^**^
**Headache n (%)**	165 (55.9%)	75 (72.8%)	9.09	0.003*
**Fatigue n (%)**	177 (60.0%)	53 (51.5%)	2.58	0.13
**Vomiting or diarrhea n (%)**	127 (43.1%)	11 (10.7%)	35.32	< 0.001**
**Fever n (%)**	274 (92.9%)	103 (100%)	7.74	0.005*
**Shock n (%)**	17 (5.7%)	70 (68.0%)	57.43	< 0.001**
**Hypoxia n (%)**	33 (11.2%)	103 (100%)	267.7	< 0.001^**^
**Acute pancreatitis manifestations**	0 (0.0%)	5 (4.8%)	14.5	< 0.001**
**Deep venous thrombosis manifestations**	5 (1.7%)	0 (0.0%)	1.77	**0.18**
**Kawasaki like diseases**	0 (0.0%)	4 (3.9%)	11.77	0.001**
**Fever duration after admission, days**				
Mean ± SD	7.39 ± 3.97	9.76 ± 4.25	U	
Median (Range)	7 (2–16)	6 (6–20)	1.39	0.16

*X* Mean, *SD* standard deviation, *U* Mann-Whitney U test, *X*^*2*^ Chi-square test

*Significant ** highly significant

Five patients manifested by acute pancreatitis. These patients presented to our emergency department by epigastric pain radiating to the back and were associated with fever, nausea, vomiting, and diarrhea. They were diagnosed with COVID-19 by nasopharyngeal reverse transcription-(PCR) before admission. Physical examination showed fever, tachycardia, and tachypnea with hypoxia (SaO2: 80-85%) on room air and scattered wheezing. Abdominal examination revealed severe epigastric tenderness. Admission laboratory workup showed elevated lipase and amylase (> 3 times of upper limit of normal level), mild increase in aspartate aminotransferase (AST) and alanine transaminase (ALT). Triglyceride levels were unremarkable. Chest and abdomen CT scans showed multifocal bilateral ground-glass opacities and normal gall bladder, biliary tract, with unremarkable pancreas respectively.

Five patients of non-severe group (1.7%) presented by deep venous thrombosis (DVT). They complained of fever and mild dry cough 3-5 days ago. On the 4th to the 6th day of the illness; these patients presented by swelling, pain, warmth, and redness in left leg or right leg or both legs. They had no history of an underlying disease, drug intake, trauma, recent surgery, or insect bite. They were diagnosed with COVID-19 by nasopharyngeal reverse transcription- (PCR) before admission. Physical examination showed fever, and mild tachypnea. Oxygen saturation was 100% on room air. On local examination, the superficial vein of the calf was dilated and there was tenderness along veins. The median value of D-dimer compared with children who did not have DVT was 2.9 μg/ml versus 0.68 μg/ml respectively and with highly significant differences (*P* < 0.001). CT angiography was performed to rule out pulmonary thromboembolism, which showed no evidence of thrombosis. Lower limb venous color Doppler ultrasound revealed dilatation and thrombosis in the external iliac and iliac veins up to the level of the bifurcation of the common iliac veins in some patients, as well as thrombosis to the superficial and small saphenous veins.

Our study reported four children with MIS-C. They presented by fever, cough, tachypnea, bilateral conjunctival injection, diffuse erythematous maculopapular rash, strawberry tongue. Physical examination showed tachycardia, tachypnea, and scattered wheezing. Respiratory distress progressed rapidly in only two patients and they were intubated due to hypoxemia (SaO2:80–85%) in room air. There were a mild increase in AST and ALT, lymphopenia, thrombocytopenia, increased D-dimer, ferritin and LDH levels. The SARS-CoV-2 RNA was detected from a nasopharyngeal swab in four patients. Coronary artery dilatation, decreased left ventricular function and mitral regurgitation reported in echocardiographic findings in only two patients. Chest CT revealed bilateral diffuse ground-glass opacities. These patients met the MIS-C definition.

Table 4 shows the underlying diseases among the studied cases; 305 patients (76.6%) had no underlying diseases while 93 patients (23.4%) had underlying diseases for example; 5.8% had epilepsy, 5.0% had immune thrombocytopenic purpura (ITP), 5.0% had chronic kidney diseases (CKD), 1.8% had neurodegenerative diseases, 1.5% had guillain barre syndrome and 1.25, 0.75, 0.5% had G6PD, beta thalassemia and dykeratosis congenita respectively.

**Table 4:** The underlying diseases among the studied cases

Studied variables	Number	(%)
**No underlying diseases**	**305**	**76.6%**
**Underlying diseases**	**93**	**23.4%**
• Epilepsy	23	5.8%
• ^*^ITP	20	5.0%
• Chronic kidney disease	20	5.0%
• Neurodegenerative disease	7	1.8%
• Arnold Chiari syndrome	7	1.8%
• Guillain barre syndrome	6	1.5%
• ^*^G6PD	5	1.25%
• Beta thalassemia	3	0.75%
• Dyskeratosis congenita	2	0.5%

*ITP* Immune thrombocytopenic purpura, *G6PD* Glucose 6 phosphate dehydrogenase deficiency

Table 5 shows laboratory data of COVID-19 children that recorded on admission according to the clinical type of severity (Group I and Group II). Abnormal findings in the two studied groups were leukopenia, lymphopenia, high CRP and ferritin levels. Some features differed significantly between the two studied groups of COVID-19, including decreased Hb level (*p* < 0.001), decreased absolute neutrophil count [ANC] (*p* < 0·001), decreased platelet count (*p* < 0.001), and high levels of ESR (*p* = 0·002) and D-dimer (*p* = 0·04).

**Table 5:** Laboratory and radiological characteristics of the COVID-19 groups

Studied variables	COVID-19 groups		Test f sig.	*P* value
	Group I *N* = 295	Group II *N* = 103		
**Hb** (11–13 g/dL)				
Mean ± SD	11.38 ± 1.86	10.46 ± 1.15	6.31	< 0.001^**^
Median (Range)	11.5 (4.5–14)	10.5 (8.2–12)		
**WBCs** (5.5–15.5 × 10^3^ / μL)				
Mean ± SD	10.91 ± 4.952	7.22 ± 5.013	3.1	0.002*
Median (Range)	4 (2.400–11.00)	4.300 (3.200–7.700)		
**ANC** (1.5–7.5 × 10^3^/μL)				
Mean ± SD	4.51 ± 1.24	4.03 ± 2.64	4.06	< 0.001**
Median (Range)	2.20 (1.0–6.30)	2.50 (2.00–4.20)		
**Lymphocytes** (1.5–8.5 × 10^3^/μL)				
Mean ± SD	2.51 ± 1.21	1.140 ± 0.51	2.13	0.03*
Median (Range)	0.90 (0.10–3.40)	1 (0.50–2.40)		
**Platelets** (150–450 × 10^3^/μL)				
Mean ± SD	210.46 ± 76.55	179.28 ± 67.37	5.13	< 0.001**
Median (Range)	212 (10–344)	191 (34–313)		
**CRP** (0–5.00 mg/L)				
Mean ± SD	76.58 ± 56.88	78.44 ± 43.53	2.15	0.03*
Median (Range)	77 (0–270)	98 (0–148)		
**ESR** (0–10 mm/hr.)				
Mean ± SD	32.52 ± 13.08	27.82 ± 13.11	3.15	0.002*
Median (Range)	35 (15–55)	25 (15–50)		
**Ferritin** (7–140 ng/ml)				
Mean ± SD	453.99 ± 405.27	438.41 ± 218.50	3.72	< 0.001**
Median (Range)	300 (95–4000)	480 (90–700)		
**D-dimer** (< 0.5 μg/ml)				
Mean ± SD	0.881 ± 0.591	1.196 ± 0.849	2.09	0.04*
Median (Range)	1.4 (1.7–3.3)	1 (1.7–5)		
**CT finding: N (%)**				
Null	235 (79.7%)	16 (15.5%)		
Ground-glass opacities	36 (12.2%)	70 (68.0%)	144.1	< 0.001^**^
Crazy paving pattern	24 (8.1%)	17 (16.5%)		

**Fig. 2:**
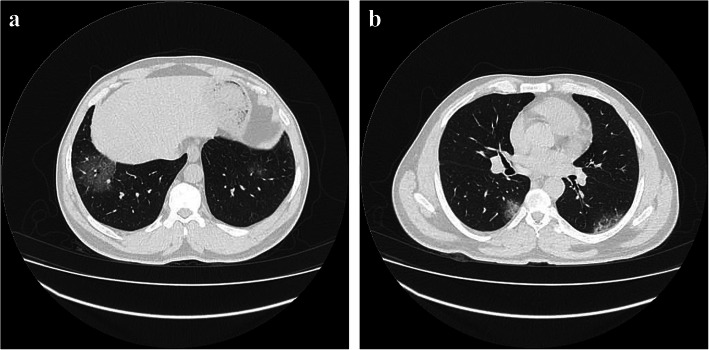
**a** Male, 14 years old. Chest CT showed bilateral basal ground glass opacities. **b** Male, 15 years old. Chest CT showed crazy paving pattern in both lungs

Regarding to CT chest findings, normal findings presented in 235 patients (79.7%) in non-severe COVID-19 group and 16 patients (15.5%) in severe COVID-19 group. Abnormal radiographic presentations were pulmonary ground-glass opacities on CT scan, suggesting pneumonia presented in 36 patients (12.2%) in non-severe COVID-19 group and 70 patients (68%) in severe COVID-19 group (Fig. 2a) and crazy paving pattern presented in 24 patients (8.1%) in non-severe COVID-19 group and 17 patients (16.5%) in severe COVID-19 group (Fig. 2b). There was a highly significant difference between the two studied groups of COVID-19 regarding to abnormal radiographic findings (*p* < 0.001) (Table 5).

As regard multivariate regression analysis for COVID-19 severity; showed that severity of COVID-19 in children was significantly higher among children with higher level of D-dimer and among those with lymphopenia (Table 6).

**Table 6:** Multivariate regression analysis of independent risk factors for predicting severe cases of COVID-19

	*P* value	OR	95% CI (lower – upper)
**Age / months**	0.14	1.07	0.58–1.99
**Sex / N (%)**	0.36	1.14	0.68–2.33
**Hb**	0.65	0.67	0.05–1.88
**ANC**	0.78	0.89	0.71–2.44
**Platelets count**	0.15	0.58	0.08–3.25
**ESR**	0.08	1.20	0.83–5.01
**D-dimer**	0.01*	2.5	1.56–9.34
**CT finding**	0.23	1.12	0.82–4.12
**Lymphopenia**	0.03*	3.1	1.89–7.81

*OR* odds ratio, *CI* confidence interval

* Significant

## Discussion

COVID-19 caused by a novel coronavirus is a highly contagious disease. Transmission occurred through an infected person or an asymptomatic carrier. The main route of transmission is respiratory droplet, contact and may be the digestive tract with incubation period from 1 to 14 days might be up to 24 days [20].

Regarding to demographic and clinical data of COVID-19 groups, most of the confirmed cases were non-severe patients (*n* = 295) (74.1%) and 103 cases (25.9%) were severe patients. In China, disease severity in children was lower with 94% having asymptomatic, mild or moderate disease, 5% having severe disease, and < 1% having critical disease [11].

Most common affected age was less than 5 years and older than 10 years, but age from 5 to 10 years was common in severe cases. Boys were more affected than girls (52% versus 48%). Most of the confirmed cases (68.6%) had a contact history to family members infected with COVID-19.

Dong et al. [11] demonstrated that boys were more affected than girls in COVID-19 outbreaks (56.6% versus 43.4%) with median age 7 years (interquartile range 2–13 years). More than 90% of patients had asymptomatic, mild, and moderate cases. Severe and critical cases were 10.6, 7.3, 4.2, 4.1, and 3.0% for the age groups, < 1, 1 to 5, 6 to 10, 11 to 15, and ≥ 16 years, respectively. This in agreement with two recent studies which showed the same epidemiological studies that boys were more affected than girls [21, 22].

Age range of COVID-19 children was 0–17 years, mean age was 5.5 years; also 58% of the patients were male. 60.1% of the patients were older than 5 years with significant difference among number of age groups. 86.4% of confirmed COVID-19 children had close contact with COVID-19 family members [23].

*Hoang* et al. [24] showed that 75.6% of patients were in contact with family members diagnosed with COVID-19.

In this current study, fever, headache, fatigue and shock were the most prominent (95, 60.3, and 57.8% and 21.8% respectively).while cough, tachypnea, hypoxia and diarrhea were (39, 41.7, 34.2 and 34.7% respectively), and both rhinorrhea and sore throat were 18.3%.

*Mantovani* et al. [25] found that 79% of patients had mild symptoms and 4% were critical, most frequent presentations were fever (47%), cough (37%), while, diarrhea, nasal congestion and dyspnea were (4, 2 and 1% respectively). *Hoang* et al. [24] reported that fever (59.1%) and cough (55.9%) were the prominent manifestations, rhinorrhea and nasal congestion in 20%, fatigue in 18.7%, sore throat in 18.2%, diarrhea in 11.7% and headache in 4.3% of affected children.

The prevalence of critical illness was 0% and the most frequent symptoms were fever (51.2%) and cough (37%) [23], some patients presented with mild symptoms as abdominal pain, decreased appetite and fatigue [26–28]*.*

Fourteen children of studied patients (3.5%) were manifested wit atypical presentations; 5 children (1.25%) manifested by picture of acute pancreatitis, 5 children (1.25%) presented by manifestations of deep venous thrombosis and 4 children (1.0%) had MIS-C.

*Alloway* et al. [29] reported necrotizing pancreatitis by abdominal ultrasound and CT in a 7-year-old girl 2 weeks prior to her diagnosis with COVID-19 infection, she was presented by anorexia, abdominal pain, fever and elevated serum lipase 1672 U/L with family history exposure. *Samies* et al. [30] reported symptoms of acute pancreatitis in 2 boys and a girl under the age of 16 years a week within their COVID-19 infection. They were diagnosed by elevated both serum amylase and lipase and CT abdomen. The pathophysiology of the pancreatic involvement by SARS-CoV-2 is due to angiotensin converting enzyme 2 expressions in both the islet cells and the exocrine portions of the pancreas [31]. During SARS-CoV-2 infection pancreatic injury might be secondary to an immune mediated injury [32].

*Visveswaran* et al. [33] reported extensive venous thrombosis, severe venous outflow obstruction with painful swollen and gangrenous limb associated with SARS-CoV-2 infection in a 12-year-old girl. Venous thromboembolism is frequent in COVID-19. DVT was detected by Doppler ultrasound in 15% of COVID-19 patients with pneumonia and elevated D-dimer [34]. Medium vessel vasculopathy was reported in some patients with MIS-C [35]. The prevalence of DVT in patients with COVID-19 admitted to intensive care was 16–49% [36].

*Verdoni* et al. [37] illustrated five patients had classic form of Kawasaki disease (non-exudative conjunctivitis, hand and feet erythema or firm induration, polymorphic rash and latero-cervical lymphadenopathy. Five patients had MIS-C, presenting with non-exudative bulbar conjunctivitis; changes of the lips or oral cavity; and polymorphic rash associated with an abnormal echocardiography.

In this current study, 41.7% of severe patients needed mechanical ventilation due to hypoxia, with the death of 20.4% (*n* = 21) of severe cases. By the end of the first week, patients with severe manifestation developed hypoxemia and hypoperfusion [37]. *Bhumbra* et al. [38] found seven (36.8%) were critically ill in ICU, and four of them (21%) required mechanical ventilation. Risk factors with critical illness were older age, longer duration of symptoms, and lower oxygen saturation at the beginning of presentation. Average of mechanical ventilation duration was 14.1 days with the death of one patient. Lower proportions of mechanically ventilated critically ill pediatric COVID-19 and fewer ventilated days have been reported by United State hospitals [39, 40]. Certain centers reported greater proportions of critically ill pediatric patient required mechanical ventilation [41, 42]. Higher proportion of our patients being in the severe category, because they have underlying diseases and low immunity and this makes their condition rapidly deteriorate and they need emergency support. So, this can explain high mortality rate of 20.4% in the severe group.

In our study, leukopenia and lymphopenia were common in children with COVID-19. Lymphopenia results from consumption of lymphocytes by SARS-CoV-2 and reflects severe disease [38] . In adults with COVID-19, up to 83.2% are lymphopenic [43, 44], 33.7% are leukopenic [8]. *Kosmeri* et al. [45] reported that leukopenia was the common findings in children with COVID-19 and lymphopenia was found in hospitalized older children. *Raba* et al. [46] found 16% of infants with COVID-19 had lymphopenia. *Ding* et al. [23] showed that 28.9% of pediatric patients with COVID-19 had leukopenia/ lymphopenia.

Our results showed high CRP in children with COVID-19 but, adults have a much higher prevalence of increased CRP than children, suggesting a much milder immunological response in children and less immune damage [47].

Erythrocyte sedimentation rate (ESR) and D-dimer were higher in the studied group with severe and critical disease. This is in agreement with studies which showed elevation of D-dimer to some extent, especially those with severe disease (LDH and D-dimer) [8, 48]. *Razavi* et al. [49] found elevation of CRP, ESR and D-dimer in children with severe manifestations. *Momtazmanesh* et al. [50] reported elevation of ESR and CRP 71.8 and 75.4% and elevation of LDH and D-dimer in 40% of patients.

Around the 7th to the 14th day of COVID-19 infection, the disease begins to affect lungs, heart, gastrointestinal tract with greater SARS-CoV-2 cell receptor expression, the angiotensin-converting enzyme 2 (ACE2) [51], with characteristic clinical symptoms and increase in the inflammatory mediators and cytokines levels [52]. During this stage of the disease, hematological changes developed, particularly a significant lymphopenia, and this may be explained by (a) direct infection in these cells, causing their lysis by SARS-CoV-2, since lymphocytes have ACE2 receptors on the surface; (b) lymphocyte apoptosis caused by the systemic inflammatory process with consequent cytokines production; (c) atrophy of lymphoid organs, such as the spleen, impairing lymphocyte turnover [53].

Our chest CT findings of children with COVID-19 were normal in 235 patients (79.7%) in non-severe COVID-19 group and 16 patients (15.5%) in severe COVID-19 group and abnormal findings as pulmonary ground-glass opacities and crazy paving patterns (68 and 16.5% respectively) which were common in severe and critical patients. *Xia* et al. [12] reported normal CT findings in more than one third of asymptomatic children and 50% of children with moderate or severe COVID-19 pulmonary disease had consolidation with a halo sign, bilateral multi-lobular diffuse ground-glass opacities and crazy-paving picture in the CT scan. *Sun* et al. [10] showed that 75% of severe case had ground glass opacities and 12.5% had white lung in their CT findings. *Ding* et al. [23] showed that 53.9% of children diagnosed with pneumonia had ground-glass opacity observed in the CT scan. In a systematic review study, 674 children with confirmed COVID-19 infection who underwent imaging, 50% had abnormalities. Among 605 children who underwent chest CT, 33% had normal findings, 29% had ground glass opacities, 27% had nonspecific unilateral findings, and 23% had bilateral findings [54].

For better clarification of COVID-19-severity in children, we found COVID-severity was associated with indicators of illness severity, including decreased ANC, higher level of D-dimer, hypoxia, shock and mechanical ventilation.

## Conclusion

Most children in our study had non- severe type of COVID-19 and children with severe type had decreased ANC, higher level of D-dimer, hypoxia, shock and mechanical ventilation. SARS-CoV-2 infection usually causes extrapulmonary manifestations out of fever and respiratory manifestations; including MIS-C, GIT, cardiovascular and hematological. So we highlight the importance for pediatricians to give thought to extrapulmonary manifestations as a differential diagnosis of SARS-CoV-2 infection in pediatric patients, especially during the COVID-19 pandemic. Consequently, laboratory tests, including serum levels of ALT, AST, creatinine, inflammatory markers, and myocardial enzymes in critically ill children or those who require oxygen who infected with SARS-CoV-2, should be considered to identify any non-pulmonary manifestations of the disease and to prevent poor prognosis.

The limitation of our cohort was the small sample size.
